# Supplementary material for: An Archaea-specific c-type cytochrome maturation machinery is crucial for methanogenesis in Methanosarcina acetivorans
Source: eLife. 2022 Apr 5;11:e76970. doi: 10.7554/eLife.76970 (PMC9084895; doi:10.7554/eLife.76970)
Supplement: Figure 1—source data 1. [file elife-76970-fig1-data1.pdf]

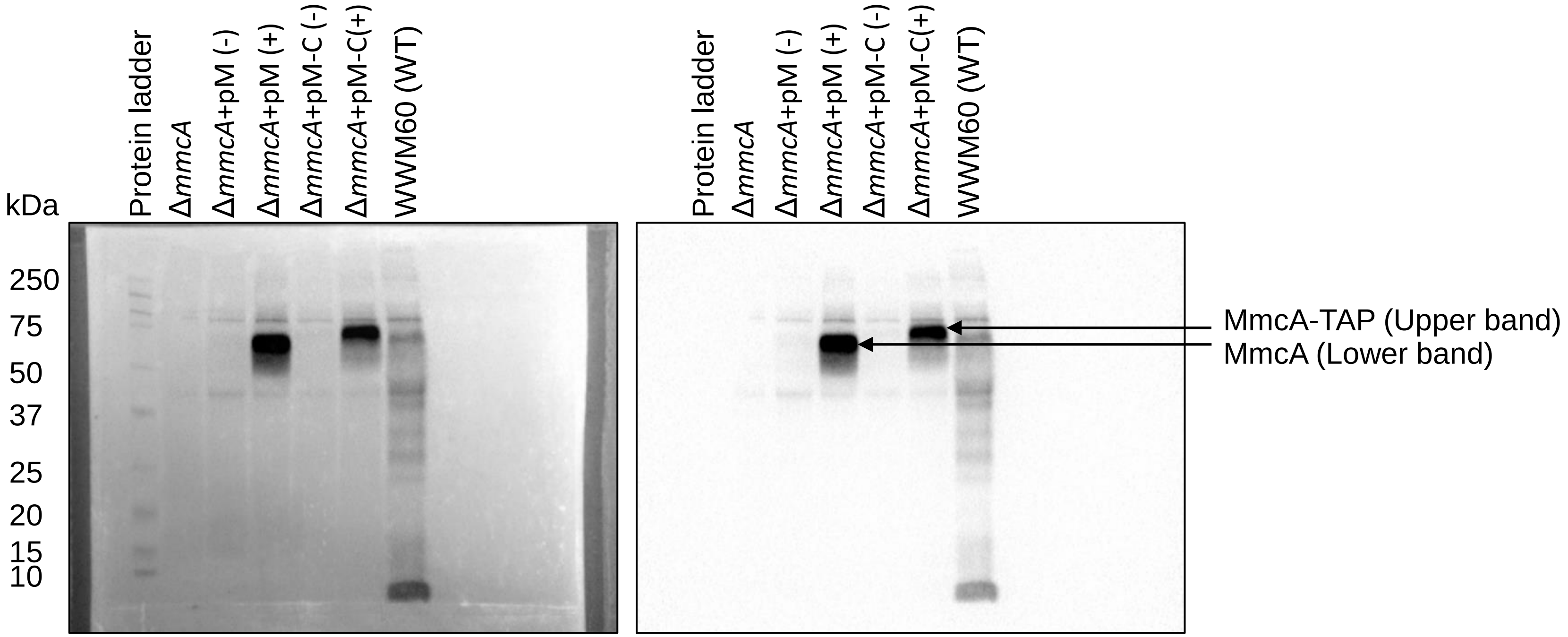

Figure 1b: Heme stain, Left hand side (image merged with ladder), Right hand side (image used in figure 1b). For further details, refer to figure legend for Figure 1b.

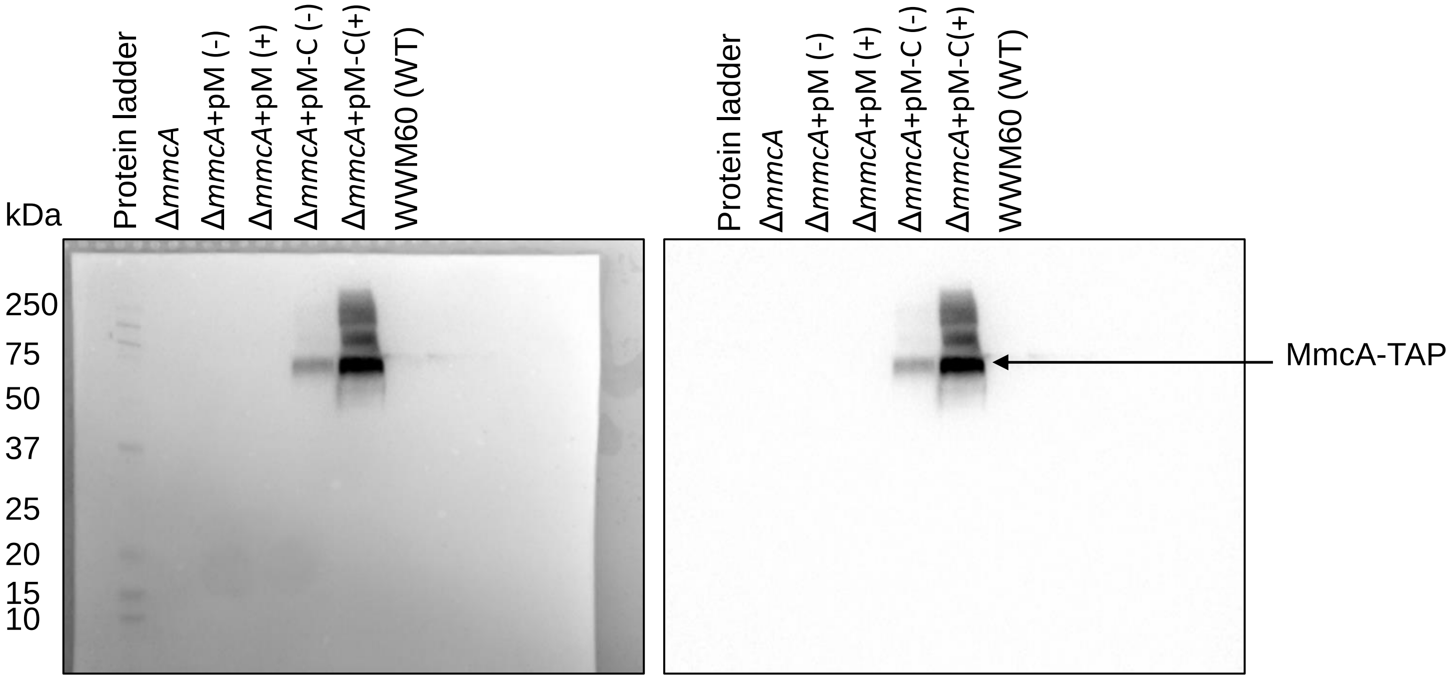

Figure 1b: anti-Flag Western Blot, Left hand side (image merged with ladder), Right hand side (image used in figure 1b). For further details, refer to figure legend for Figure 1b.
