# Supplementary figures and images for: An Archaea-specific c-type cytochrome maturation machinery is crucial for methanogenesis in Methanosarcina acetivorans
Source: eLife. 2022 Apr 5;11:e76970. doi: 10.7554/eLife.76970 (PMC9084895; doi:10.7554/eLife.76970)

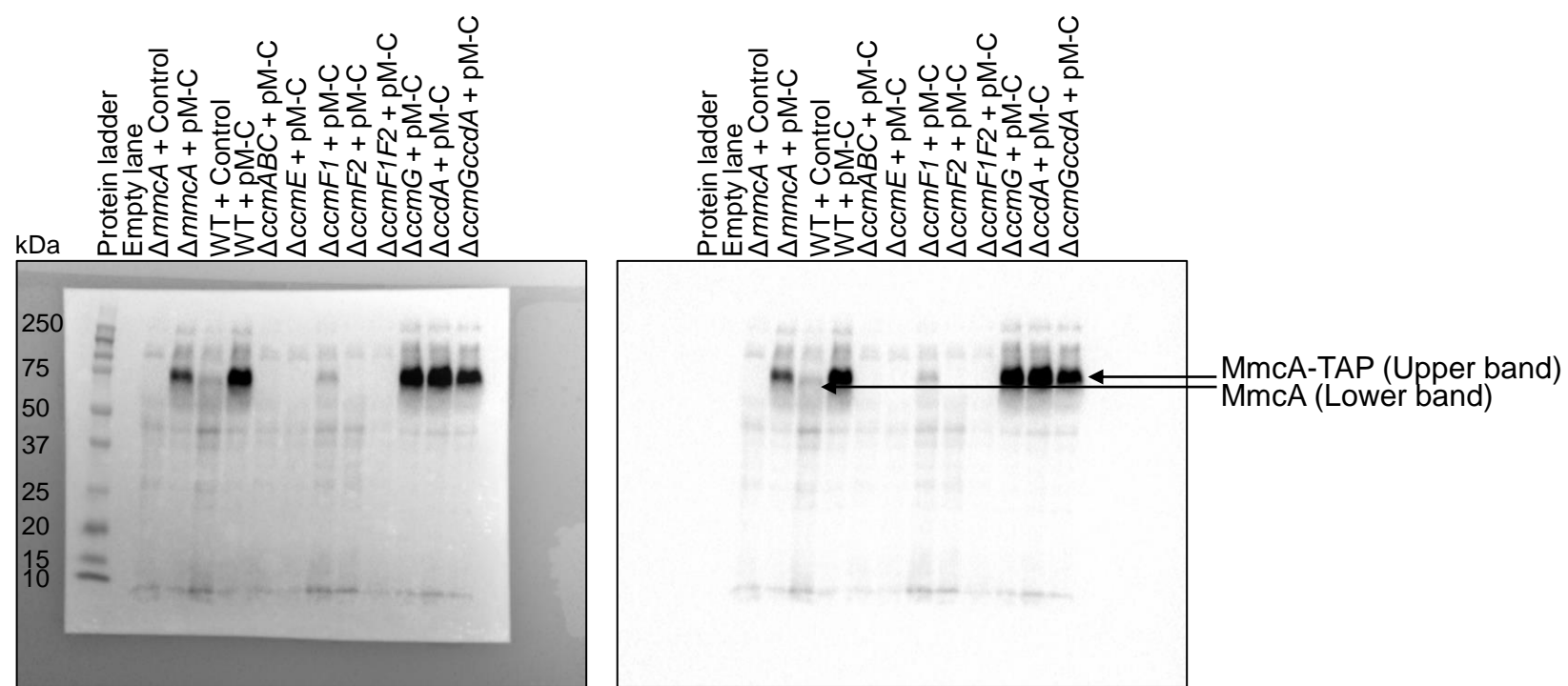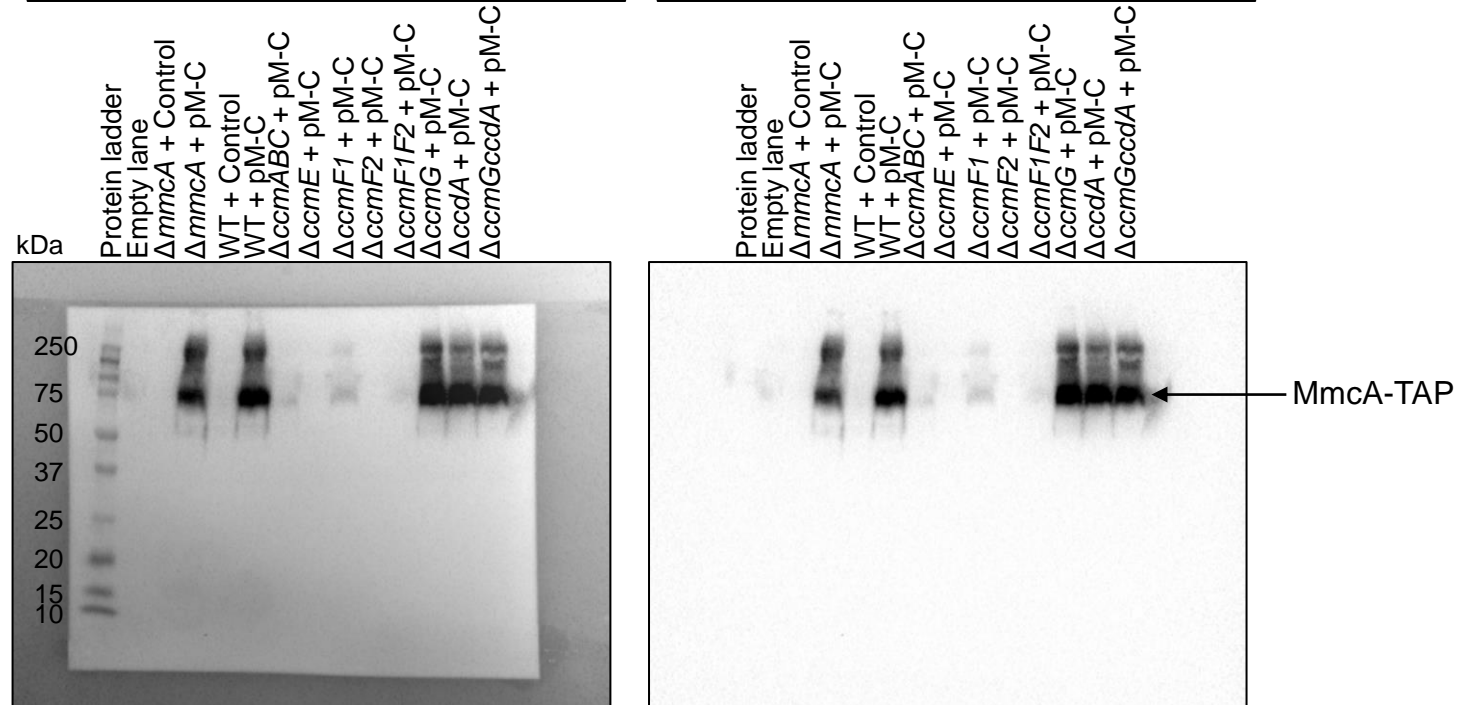

Supplement: Figure 2—source data 1. [file elife-76970-fig2-data1.pdf]
