# Supplementary material for: An Archaea-specific c-type cytochrome maturation machinery is crucial for methanogenesis in Methanosarcina acetivorans
Source: eLife. 2022 Apr 5;11:e76970. doi: 10.7554/eLife.76970 (PMC9084895; doi:10.7554/eLife.76970)
Supplement: Figure 3—figure supplement 1—source data 1. [file elife-76970-fig3-figsupp1-data1.pdf]

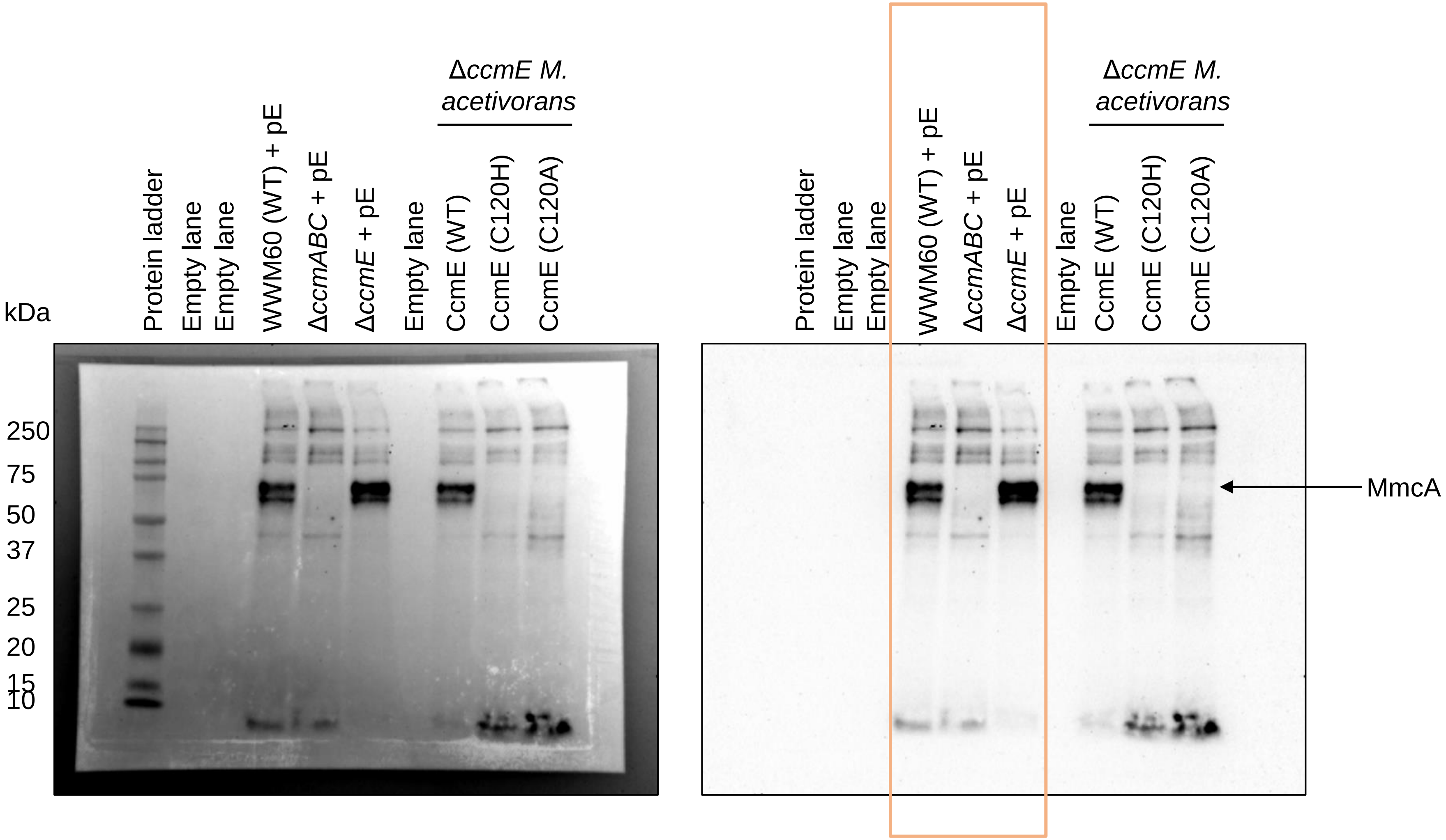

Figure 3-figure supplement 1-source data 1: Heme stain, Left hand side (image merged with ladder), Right hand side (image used for Figure 3-figure supplement 1 and Figure 4-figure supplement 2). Lanes in **orange box** were used in Figure 3-figure supplement 1. For detail, refer to the figure legend for Figure 3-figure supplement 1.
