# Supplementary material for: An Archaea-specific c-type cytochrome maturation machinery is crucial for methanogenesis in Methanosarcina acetivorans
Source: eLife. 2022 Apr 5;11:e76970. doi: 10.7554/eLife.76970 (PMC9084895; doi:10.7554/eLife.76970)
Supplement: Figure 4—source data 1. [file elife-76970-fig4-data1.pdf]

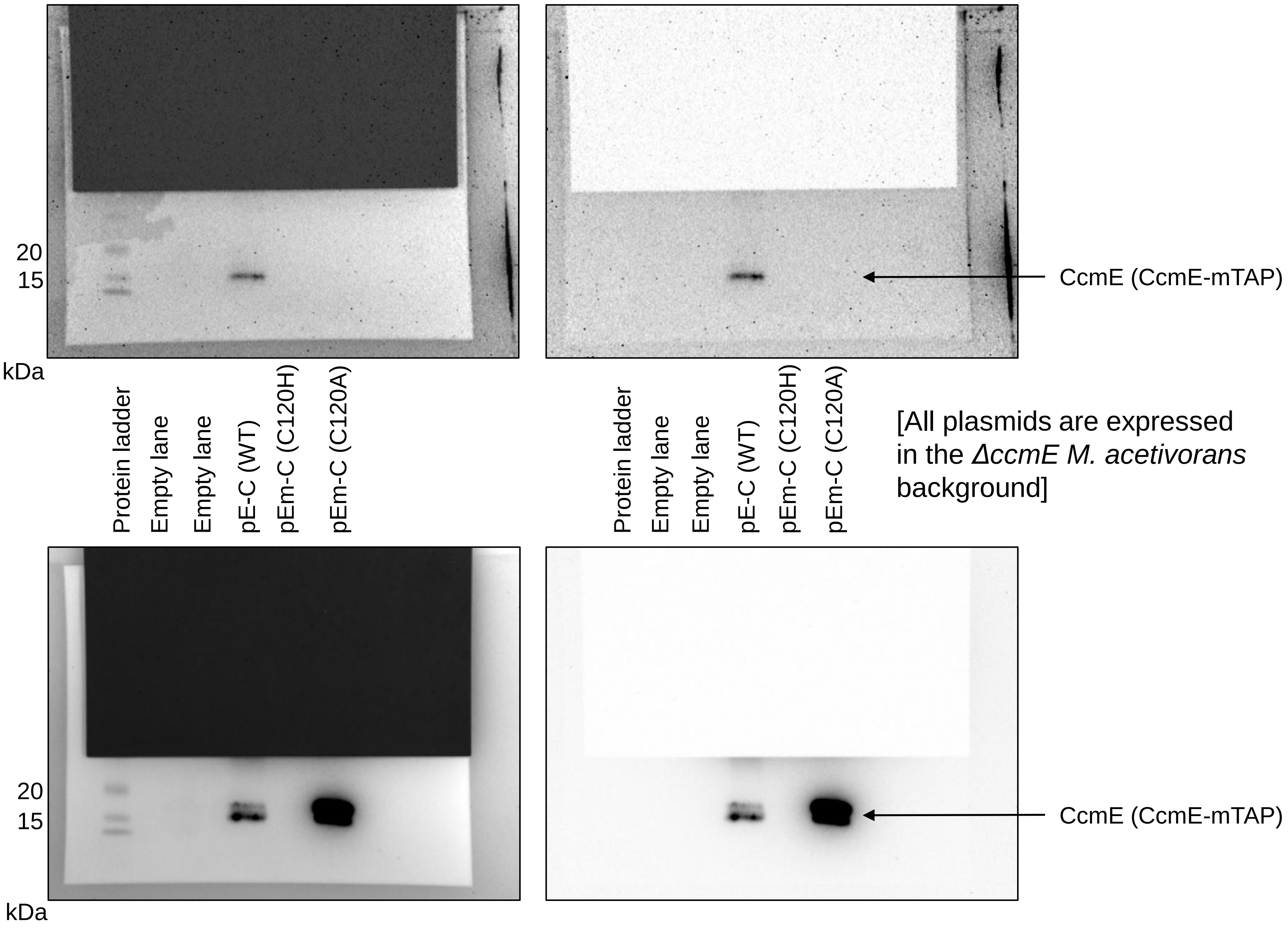

Figure 4c: Heme stain, Left hand side (image merged with ladder), Right hand side (image used in figure 4c). For details, refer to the legend for Figure 4c.

Figure 4c: anti-Flag Western Blot, Left hand side (image merged with ladder), Right hand side (image used in figure 4c). For details, refer to the legend for Figure 4c.

Note: The labels are the same for gels and blots on the top and bottom
