# Supplementary material for: An Archaea-specific c-type cytochrome maturation machinery is crucial for methanogenesis in Methanosarcina acetivorans
Source: eLife. 2022 Apr 5;11:e76970. doi: 10.7554/eLife.76970 (PMC9084895; doi:10.7554/eLife.76970)
Supplement: Figure 4—figure supplement 2—source data 1. [file elife-76970-fig4-figsupp2-data1.pdf]

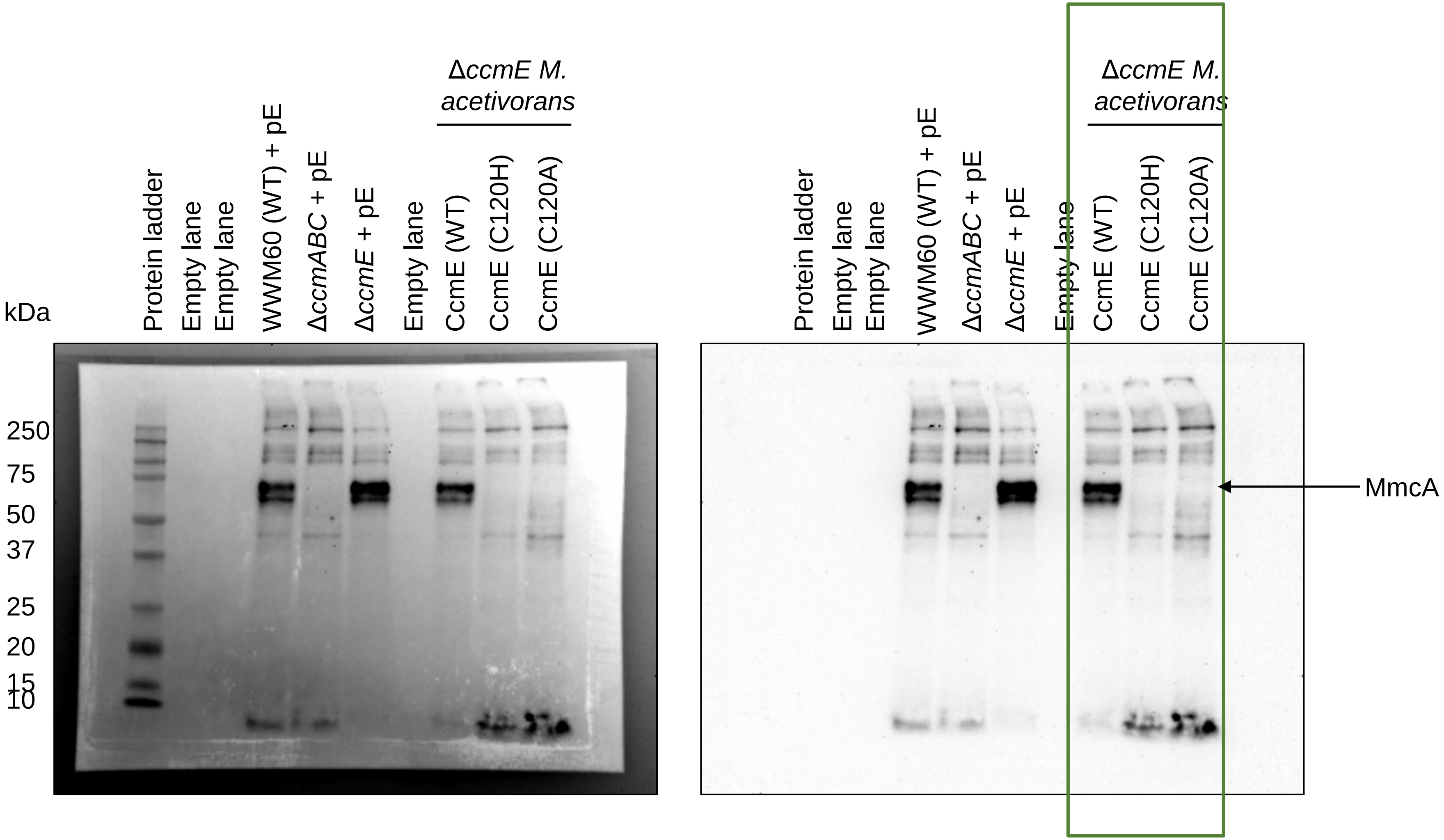

Figure 4-figure supplement 2: Heme stain, Left hand side (image merged with ladder), Right hand side (image used for Figure 3-figure supplement 1 and Figure 4-figure supplement 2). Lanes in **green box** were used in Figure 4-figure supplement 2. For detail, refer to the figure legend for Figure 4-figure supplement 2.
