# Supplementary material for: An Archaea-specific c-type cytochrome maturation machinery is crucial for methanogenesis in Methanosarcina acetivorans
Source: eLife. 2022 Apr 5;11:e76970. doi: 10.7554/eLife.76970 (PMC9084895; doi:10.7554/eLife.76970)
Supplement: Figure 5—source data 1. [file elife-76970-fig5-data1.pdf]

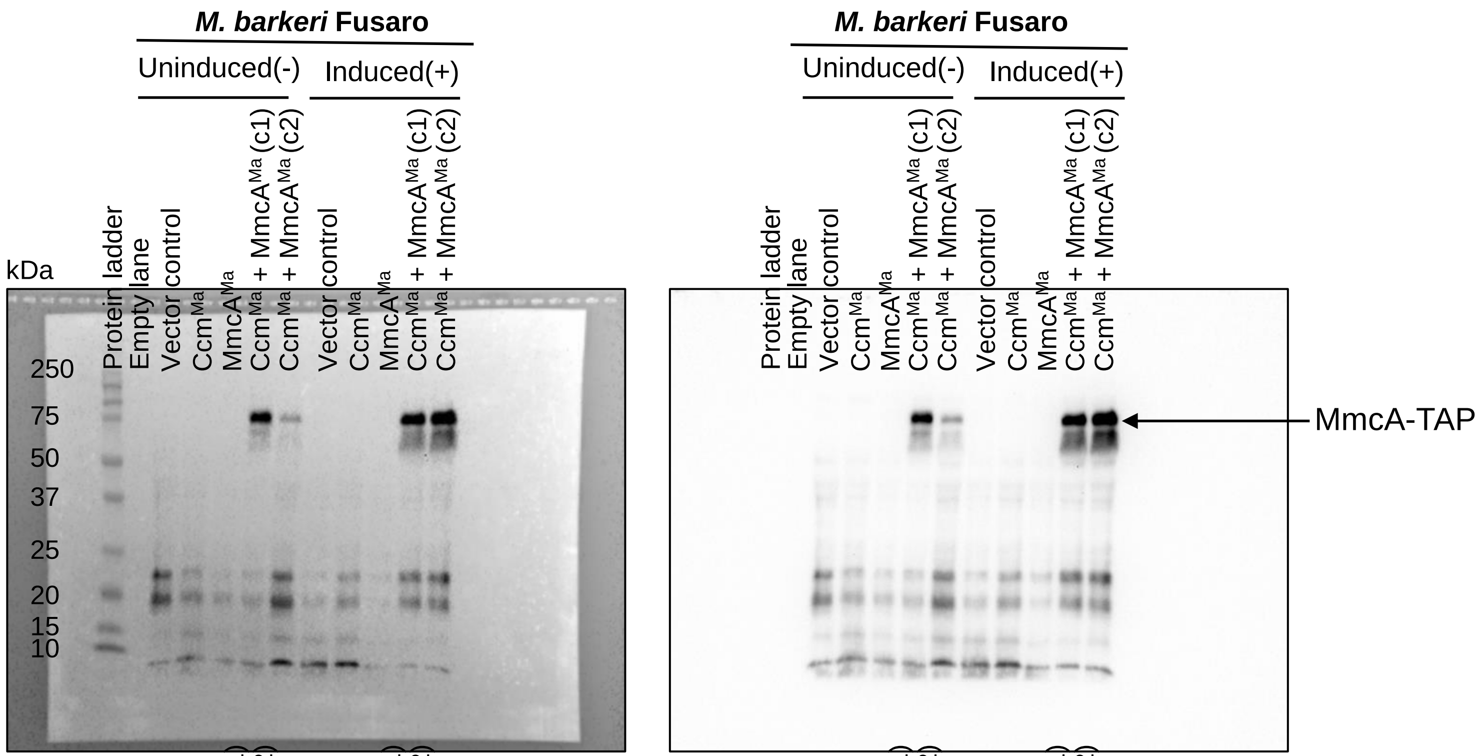

Figure 5b: Heme stain, Left hand side (image merged with ladder), Right hand side (image used in figure 5b). Data from induced cultures were used in Figure 5b. For details, refer to the legend for Figure 5b.

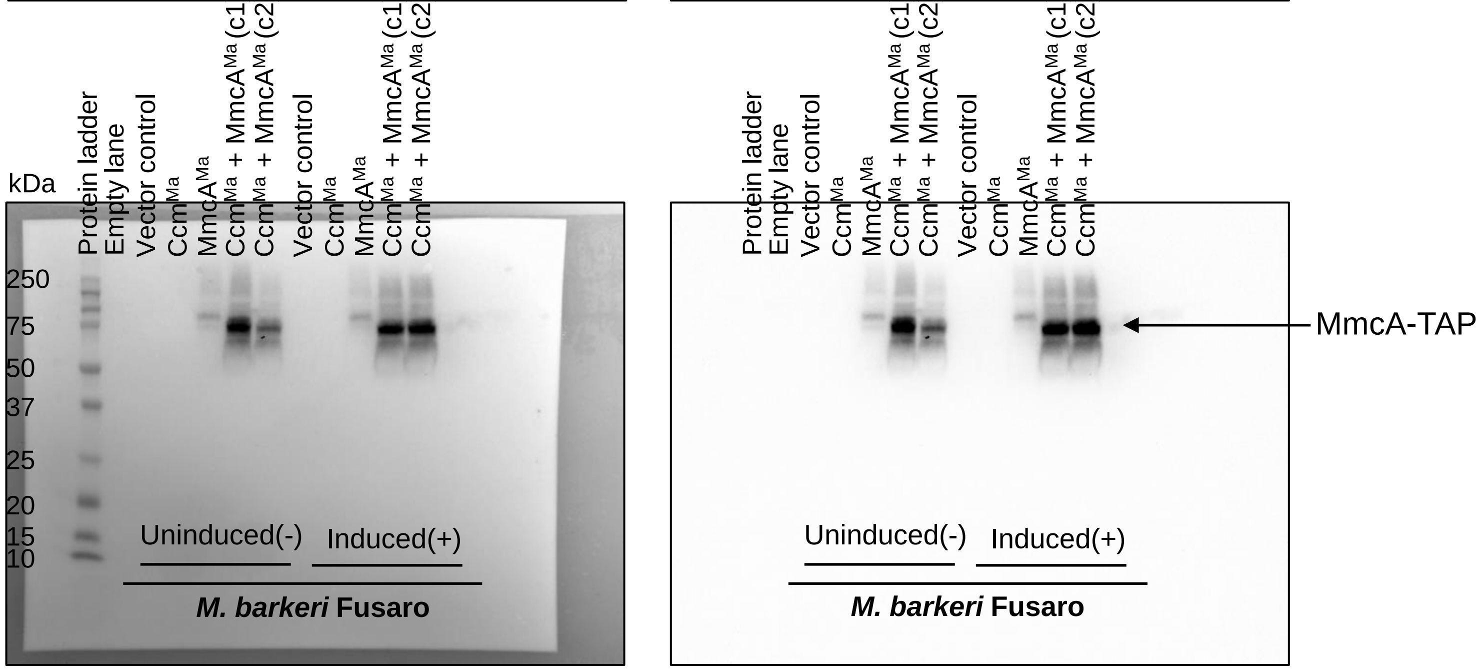

Figure 5b: anti-Flag Western Blot, Left hand side (image merged with ladder), Right hand side (image used in figure 5b). Data from induced cultures were used in Figure 5b. For details, refer to the legend for Figure 5b.
