# Supplementary material for: An Archaea-specific c-type cytochrome maturation machinery is crucial for methanogenesis in Methanosarcina acetivorans
Source: eLife. 2022 Apr 5;11:e76970. doi: 10.7554/eLife.76970 (PMC9084895; doi:10.7554/eLife.76970)
Supplement: Figure 5—figure supplement 1—source data 1. [file elife-76970-fig5-figsupp1-data1.pdf]

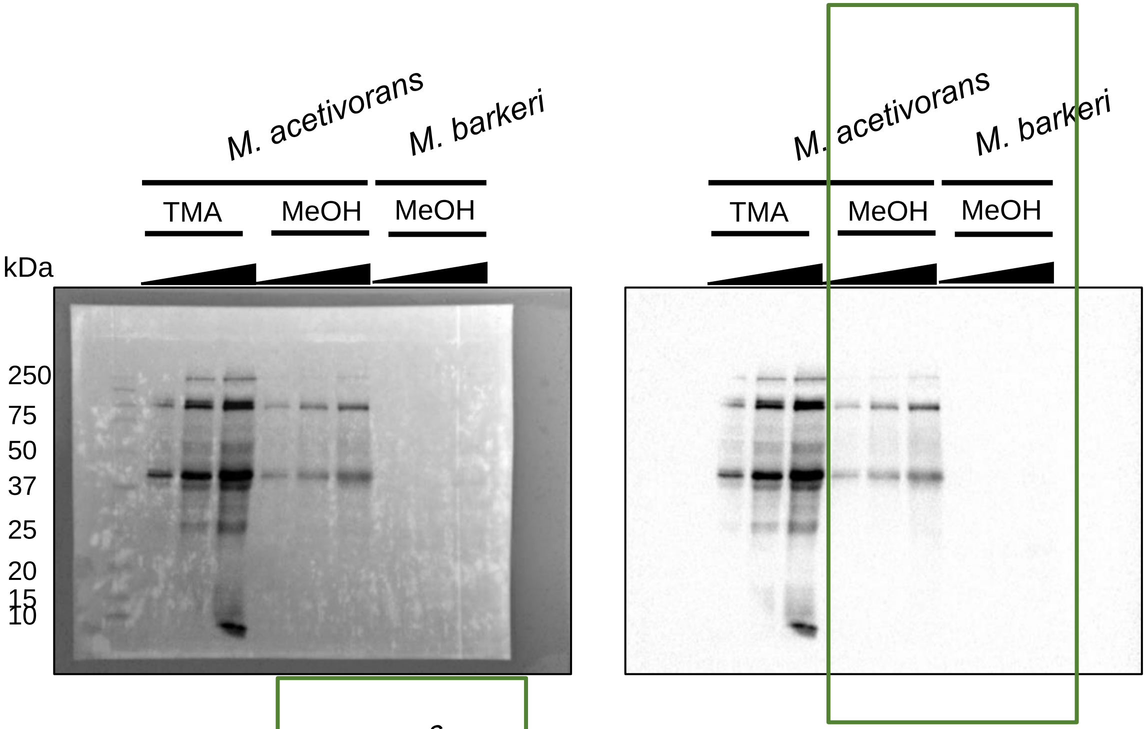

Figure 5-figure supplement 1: Heme stain, Left hand side (image merged with ladder), Right hand side (image used in Figure 5-figure supplement 1). Only the result comparing *M. acetivorans* and *M. barkeri* grown in methanol (MeOH) was included (shown here in green box) in Figure 5-figure supplement 1. For details, refer to the figure legend for Figure 5-figure supplement 1.

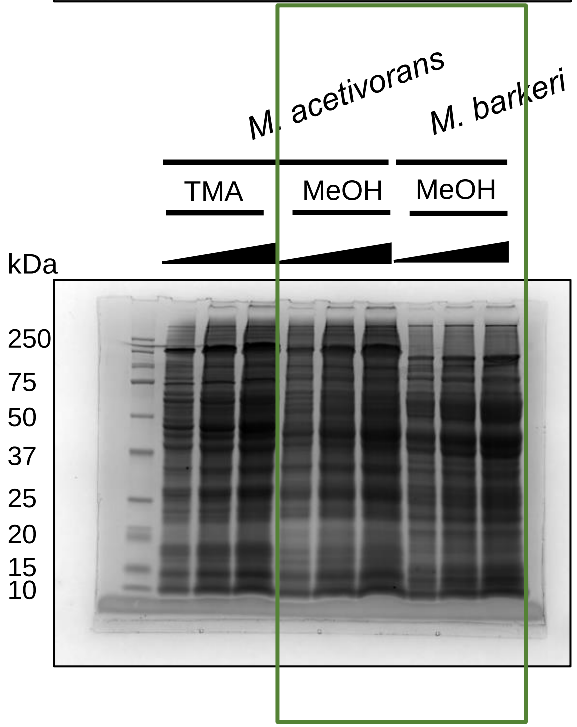

Figure 5-figure supplement 1 : Coomassie gels for Heme stains shown above. Only the result comparing *M. acetivorans* and *M. barkeri* grown in methanol (MeOH) was included (shown here in green box) in Figure 5-figure supplement 1. For details, please refer to the figure legend for Figure 5-figure supplement 1.
