# Supplementary material for: An Archaea-specific c-type cytochrome maturation machinery is crucial for methanogenesis in Methanosarcina acetivorans
Source: eLife. 2022 Apr 5;11:e76970. doi: 10.7554/eLife.76970 (PMC9084895; doi:10.7554/eLife.76970)
Supplement: Supplementary file 1. [file elife-76970-supp1.docx]

| **Position** | **Mutation** | **Notes** |
| --- | --- | --- |
| 487691 | C added | Present in Parent (WWM60) |
| 836182-836771 | ∆*hpt*::P*mcrB*-*tetR* | Present in Parent (WWM60) |
| 941168 | A5->6 | Present in Parent (WWM60) |
| 1314120 | Δ1bp | Present in Parent (WWM60) |
| **1698001** | **∆2684 bp** | **∆*ccmABC* (only present in DDN029)** |
| 2086881-82 | TC->CT | Present in Parent (WWM60) |
| 2086886 | G->T | Present in Parent (WWM60) |
| 2534543 | C added | Present in Parent (WWM60) |
| 2836646 | A->G (F64L) | Present in Parent (WWM60) |
| 2867059 | T->G (H313Q) | Present in Parent (WWM60) |
| 3433201 | Δ1bp | Present in Parent (WWM60) |
| 4295452 | Δ1bp | Present in Parent (WWM60) |
| 4874567 | Δ1bp | Present in Parent (WWM60) |
| 4945345 | C added | Present in Parent (WWM60) |
| 5078585 | G->A (M1M) | Present in Parent (WWM60) |

**Supplementary Table 1:** List of mutations in CRISPR-edited mutant strain DDN029 containing a ∆ *ccmABC* in-frame deletion mutation
