# Supplementary material for: An Archaea-specific c-type cytochrome maturation machinery is crucial for methanogenesis in Methanosarcina acetivorans
Source: eLife. 2022 Apr 5;11:e76970. doi: 10.7554/eLife.76970 (PMC9084895; doi:10.7554/eLife.76970)
Supplement: Supplementary file 2. [file elife-76970-supp2.docx]

**Supplementary Table 2:** Growth data of *M. acetivorans* strains shown in Figure 6

| *M. acetivorans* strains | Doubling time (Td) (hrs) | Max.OD_600_ (OD^M^) | P value for WT vs mutants (Td/OD^M^) | P value for Δ*mmcA* vs Δ*ccm* mutants (Td/OD^M^) | P value for Δ*ccmABC* vs Δ*ccmE* mutants (Td/OD^M^) |
| --- | --- | --- | --- | --- | --- |
| Trimethylamine (TMA) as a growth substrate | | | | | |
| WWM60 (WT) | 12.13 ± 0.04 | 1.29 ± 0.01 |  |  |  |
| Δ*mmcA* | 19.89 ± 0.44 | 1.14 ± 0.02 | 3.73E-05 (***)/  0.0013 ((**) |  |  |
| Δ*ccmABC* | 20.08 ± 0.37 | 1.15 ± 0.02 | 2.70E-05 (***)/  0.0011 (**) | 0.4661 (NS)/  0.1832 (NS) |  |
| Δ*ccmE* | 20.19 ± 0.15 | 1.15 ± 0.01 | 2.21E-06 (***)/  0.0002 (**) | 0.2523 (NS)/  0.5187 (NS) | 0.3856 NS)/  0.8431 (NS) |
| Methanol (MeOH) as a growth substrate | | | | | |
| WWM60 (WT) | 10.11 ± 0.23 | 1.13 ± 0.00 |  |  |  |
| Δ*mmcA* | 27.23 ± 0.28 | 0.92 ± 0.00 | 6.66E-07 (***)/  8.30E-07(***) |  |  |
| Δ*ccmABC* | 29.40 ± 0.58 | 0.90 ± 0.01 | 1.89E-05 (***)/  2.28E-05 (***) | 0.0131(*)/  0.0477(*) |  |
| Δ*ccmE* | 29.52 ± 0.28 | 0.90 ± 0.01 | 5.85E-08 (***)/  4.71E-06 (***) | 6.06E-05 (***)/  0.0036(**) | 0.7980 (NS)/  0.0749(NS) |
| Acetate as a growth substrate | | | | | |
| WWM60 (WT) | 77.93 ± 4.32 | 0.22 ± 0.01 |  |  |  |
| Δ*mmcA* | No growth observed^#^ | - | - |  |  |
| Δ*ccmABC* | No growth observed^#^ | - | - | - |  |
| Δ*ccmE* | No growth observed^#^ | - | - | - | - |
| Dimethyl sulfide (DMS) as a growth substrate | | | | | |
| WWM60 (WT) | 32.6 ± 0.55 | 0.35 ± 0.01 |  |  |  |
| Δ*mmcA* | 53.7 ± 5.66 | 0.26 ± 0.02 | 0.0247 (*)/  0.0053(**) |  |  |
| Δ*ccmABC* | 51.1 ± 2.84 | 0.33 ± 0.00 | 0.0093 (**)/  0.0474 (*) | 0.2515 (NS)/  0.0248 (*) |  |
| Δ*ccmE* | 41.6 ± 1.68 | 0.36 ± 0.00 | 0.0055 (**)/  0.4266 (NS) | 0.0876 (NS)/  0.0215 (*) | 0.0568 (NS)/  0.0148 (*) |

All data represent the mean ± standard deviation of at least 3 biological replicates at 37 °C

# No growth observed at 37 °C for 50 days

P > 0.05 (non-significant, NS); P ≤ 0.05 (*); P ≤ 0.01 (**); P ≤ 0.0001 (***) using a two-sided Student’s t-test
