# Supplementary material for: An Archaea-specific c-type cytochrome maturation machinery is crucial for methanogenesis in Methanosarcina acetivorans
Source: eLife. 2022 Apr 5;11:e76970. doi: 10.7554/eLife.76970 (PMC9084895; doi:10.7554/eLife.76970)
Supplement: Supplementary file 3. [file elife-76970-supp3.docx]

**Supplementary Table 3:** List of target sequences used in this study

| **Gene (locus tag)** | **Target sequence (+ PAM)** | **Location on *M. acetivorans* chromosome** |
| --- | --- | --- |
| *mmcA* (MA0658) | AGTTACAGCAATACTCTTCA**CGG**  TGAATACCCAAGAGAAGAAT**AGG** | 776339 – 776358 (- strand)  774977 – 774996 (- strand) |
| *ccmABC* (MA1428-1430) | AAAAGAGGGTCAAAAAGAGC**AGG**  AGGTCAAGGGCTTTCAGGAC**AGG** | 1698029 – 1698048 (+ strand)  1700643 – 1700662 (+ strand) |
| *ccmF_2_* (MA3304) | CAGGCAGGACAGGAAATTTC**TGG**  GTCCCTGATTCCCTGGCTTA**CGG** | 4078616 – 4078635 (- strand)  4077905 – 4077924 (- strand) |
| *ccmF_1_* (MA3305) | CACAGAGACTGTAGCAAATA**TGG**  TCTCCCGGAAACGGCCTTTC**CGG** | 4079700 – 4079719 (+ strand)  4078,828 – 4078847 (- strand) |
| *ccmE* (MA4149) | TGGTGTGACATCATTTGAAC**TGG** | 5066266 - 5066285 (+ strand) |
| *ccmG* (MA4254) | GCATCTGTAATCTTTACGGC**AGG** | 5177988 – 5178007 (+ strand) |
| *ccdA* (MA4255) | TCTCCTTGTAGGGCTCTGGA**TGG** | 5179184 – 5179203 (+ strand) |
