# Supplementary material for: An Archaea-specific c-type cytochrome maturation machinery is crucial for methanogenesis in Methanosarcina acetivorans
Source: eLife. 2022 Apr 5;11:e76970. doi: 10.7554/eLife.76970 (PMC9084895; doi:10.7554/eLife.76970)
Supplement: Supplementary file 4. [file elife-76970-supp4.docx]

**Supplementary Table 4:** List of plasmids used in this study

| Plasmid | Features | Source |
| --- | --- | --- |
| pAMG40 | Vector for fosmid retrofitting that contains pC2A and λattB | (Guss et al. 2008) |
| pJK027A | Vector with P*mcrB*(*tetO1*) promoter fusion to *uidA* that contains φC31-attB and λattP | (Guss et al. 2008) |
| pJK029A | Vector with P*mcrB*(*tetO4*) promoter fusion to *uidA* that contains φC31-attB and λattP | (Guss et al. 2008) |
| pDN201 | pJK027A-derived plasmid with P*mcrB*(*tetO1*) promoter fusion to Spy *cas9* | (Nayak and Metcalf, 2017) |
| pDN401 | pDN201-derived plasmid with synthetic fragments containing P*mtaCB1* promoter fusion to sgRNAs in tandem targeting the *mmcA (MA0658)* coding sequence (CDS) | This study |
| pDN402 | pDN401-derived plasmid containing a repair template to generate an in-frame deletion of the *mmcA (MA0658)* CDS | This study |
| pDN403 | Cointegrate of pDN401 and pAMG40 | This study |
| pDN404 | Cointegrate of pDN402 and pAMG40 | This study |
| pDN406 | pJK029A-derived plasmid with P*mcrB*(*tetO4*) promoter fusion to *mmcA (MA0658)* CDS from *M. acetivorans* with a C-terminal tandem affinity purification tag (containing a 3X FLAG tag and a Twin-Strep tag) | This study |
| pDN409 | Cointegrate of pDN406 and pAMG40 | This study |
| pDN411 | pDN201-derived plasmid with synthetic fragments containing P*mtaCB1* promoter fusion to sgRNAs in tandem targeting the *ccmE* (MA4149) coding sequence (CDS) | This study |
| pDN412 | pDN411-derived plasmid containing a repair template to generate an in-frame deletion of the *ccmE* (MA4149*)* CDS | This study |
| pDN413 | Cointegrate of pDN411 and pAMG40 | This study |
| pDN414 | Cointegrate of pDN412 and pAMG40 | This study |
| pDN421 | pDN201-derived plasmid with synthetic fragments containing P*mtaCB1* promoter fusion to sgRNAs in tandem targeting the *ccmF1* (MA3305) coding sequence (CDS) | This study |
| pDN422 | pDN201-derived plasmid with synthetic fragments containing P*mtaCB1* promoter fusion to sgRNAs in tandem targeting the *ccmF2* (MA3304) coding sequence (CDS) | This study |
| pDN423 | pDN201-derived plasmid with synthetic fragments containing P*mtaCB1* promoter fusion to sgRNAs in tandem targeting the *ccmF1F2* (MA3305-3304) coding sequences (CDS) | This study |
| pDN424 | pDN421-derived plasmid containing a repair template to generate an in-frame deletion of the *ccmF1* (MA3305) CDS | This study |
| pDN425 | pDN422-derived plasmid containing a repair template to generate an in-frame deletion of the *ccmF2* (MA3304) CDS | This study |
| pDN426 | pDN423-derived plasmid containing a repair template to generate an in-frame deletion of the *ccmF1F2* (MA3305-3304) CDS | This study |
| pDN427 | Cointegrate of pDN421 and pAMG40 | This study |
| pDN428 | Cointegrate of pDN422 and pAMG40 | This study |
| pDN429 | Cointegrate of pDN423 and pAMG40 | This study |
| pDN430 | Cointegrate of pDN424 and pAMG40 | This study |
| pDN431 | Cointegrate of pDN425 and pAMG40 | This study |
| pDN432 | Cointegrate of pDN426 and pAMG40 | This study |
| pDN443 | pDN201-derived plasmid with synthetic fragments containing P*mtaCB1* promoter fusion to sgRNAs in tandem targeting the *ccmABC* (MA1428-1430) coding sequences (CDS) | This study |
| pDN444 | pDN443-derived plasmid containing a repair template to generate an in-frame deletion of the *ccmABC* (MA1428-1430) CDS | This study |
| pDN445 | Cointegrate of pDN443 and pAMG40 | This study |
| pDN446 | Cointegrate of pDN444 and pAMG40 | This study |
| pDPG001 | pDN201-derived plasmid with synthetic fragments containing P*mtaCB1* promoter fusion to sgRNAs in tandem targeting the *ccmG (MA4254)* coding sequence (CDS) | This study |
| pDPG002 | pDN201-derived plasmid with synthetic fragments containing P*mtaCB1* promoter fusion to sgRNAs in tandem targeting the *ccdA (MA4255)* coding sequence (CDS) | This study |
| pDPG003 | pDN201-derived plasmid with synthetic fragments containing P*mtaCB1* promoter fusion to sgRNAs in tandem targeting the *ccmG-ccdA (MA4254-4255)* coding sequences (CDS) | This study |
| pDPG004 | pDPG001-derived plasmid containing a repair template to generate an in-frame deletion of the *ccmG* (MA4254) CDS | This study |
| pDPG005 | pDPG002-derived plasmid containing a repair template to generate an in-frame deletion of the *ccdA* (MA4255) CDS | This study |
| pDPG006 | pDPG003-derived plasmid containing a repair template to generate an in-frame deletion of the *ccmG-ccdA* (MA4254-4254) CDS | This study |
| pDPG007 | Cointegrate of pDPG004 and pAMG40 | This study |
| pDPG008 | Cointegrate of pDPG005 and pAMG40 | This study |
| pDPG009 | Cointegrate of pDPG006 and pAMG40 | This study |
| pDPG010 | Cointegrate of pJK029A and pAMG40 | This study |
| pDPG014 | pJK029A-derived plasmid with P*mcrB*(*tetO4*) promoter fusion to *ccmABC* (MA1428-1430)*-ccmE* (MA4149)-*ccmF1F2*(MA3305-3304) CDS from *M. acetivorans* | This study |
| pDPG015 | pJK029A-derived plasmid with *M. barkeri* P*serC*(constitutive) promoter fusion to *mmcA* (MA0658*)* CDS from *M. acetivorans* with a C-terminal tandem affinity purification tag (containing a 3X FLAG tag and a Twin-Strep sequence) | This study |
| pDPG017 | pJK029A-derived plasmid with P*mcrB*(*tetO4*) promoter fusion to *ccmABC* (MA1428-1430)*-ccmE* (MA4149)-*ccmF1F2*(MA3305-3304) CDS from *M. acetivorans* and *M. barkeri* P*serC*(constitutive) promoter fusion to *mmcA* (MA0658) CDS from *M. acetivorans* with a C-terminal tandem affinity purification tag (containing a 3X FLAG tag and a Twin-Strep sequence) | This study |
| pKES30 | pJK029A-derived plasmid with P*mcrB*(*tetO4*) promoter fusion to *ccmE* (MA4149) CDS from *M. acetivorans* with a C-terminal tandem affinity purification tag (containing a 1X Strep and a 1X FLAG sequence) | This study |
| pKES031 | Cointegrate of pKES30 and pAMG40 | This study |
| pKES038 | pJK029A-derived plasmid with P*mcrB*(*tetO4*) promoter fusion to *ccmE* (MA4149) CDS with C120H substitution with a C-terminal tandem affinity purification tag (containing a 1X Strep and a 1X FLAG sequence) | This study |
| pKES039 | Cointegrate of pKES38 and pAMG40 | This study |
| pKES040 | pJK029A-derived plasmid with P*mcrB*(*tetO4*) promoter fusion to *ccmE* (MA4149) CDS with C120A substitution with a C-terminal tandem affinity purification tag (containing a 1X Strep and a 1X FLAG sequence) | This study |
| pKES041 | Cointegrate of pKES40 and pAMG40 | This study |

Note: pDN201/pJK029A derived plasmids contain the chloramphenicol resistance marker, pAMG40 contains kanamycin resistance marker, and the co-integrates of pDN201/pJK029A derived plasmids and pAMG40 contain both contain chloramphenicol and kanamycin resistance markers.
