# Supplementary material for: An Archaea-specific c-type cytochrome maturation machinery is crucial for methanogenesis in Methanosarcina acetivorans
Source: eLife. 2022 Apr 5;11:e76970. doi: 10.7554/eLife.76970 (PMC9084895; doi:10.7554/eLife.76970)
Supplement: Supplementary file 5. [file elife-76970-supp5.docx]

**Supplementary Table 5**: List of primers used in this study

| **Primer Name** | **Primer Sequence**  **(Overhangs for Gibson assembly are shown in red; sequences to introduce C120A or C120H mutations in *ccmE* are shown in purple)** |
| --- | --- |
| *mtaBC1*-f for pDPG001, 002, 003 | **TCGAAGTTTAAACCTGCAGG**GAATTACACGATCACTAATTTT |
| *mtaBC1*-sgRNA-r for pDPG001 | **GCCGTAAAGATTACAGATGC**ACATGTGCTTACAGCATAAT |
| sgRNA-scaffold-f for pDPG001 | **GCATCTGTAATCTTTACGGC**GTTTTAGAGCTAGAAATAGCAAGTTAAAATAAG |
| sgRNA-scaffold-r for pDPG001 | **TAAGCGGCCGCGATCGCCGG**TACATGAGGGCTGAAAAGCCG |
| *mtaBC1*-sgRN-r for pDPG002 | **TCCAGAGCCCTACAAGGAGA**ACATGTGCTTACAGCATA |
| sgRNA-scaffold-f for pDPG002 | **TCTCCTTGTAGGGCTCTGGA**GTTTTAGAGCTAGAAATAGCAAGTTAAAATAAG |
| sgRNA-scaffold-r for pDPG002 | **TCCAGAGCCCTACAAGGAGA**TTCGTTTTCTCTTTTTAGGTGACTG |
| pDPG004-us-f | **GCCTTTTTTTTTCGAAGTTT**CAGAAAAGAGTTTTTGAAAGTGCAGC |
| pDPG004-us-r1 | **AGTAAAGAACGGCTCTTTC**CAGGATTAGTGGTATAATTAGTTTATTC |
| pDPG004-ds-f | **TAATTATACCACTAATCCTG**GAAAGAGCCGTTCTTTACTATGAG |
| pDPG004-ds-r | **TGTAATTCCCTGCAGGTTT**TACAGTAAGAACTATGAGTCAGGAG |
| pDPG005-us-f | **GCCTTTTTTTTTCGAAGTTT**CCCTGAATGCAGAGCTGAAA |
| pDPG005-us-r | **TGAGGTGGTTCTGATAGGC**AACCACGGGTGAAATAGCTTC |
| pDPG005-ds-f1 | **AAGCTATTTCACCCGTGGTT**GCCTATCAGAACCACCTCAG |
| pDPG005-ds-r | **TGTAATTCCCTGCAGGTTT**CTTTCATGTTTAGGGGACCTGC |
| pDPG006-us-r2 | **TGAGGTGGTTCTGATAGGC**CAGGATTAGTGGTATAATTAGTTTATTCATG |
| pDPG006-ds-f2 | **TAATTATACCACTAATCCTG**GCCTATCAGAACCACCTCAGC |
| pDPG004_seq-f | AATTCCTACCTTCAGGCTCAAG |
| pDPG004_seq-r | TGACGCAGGATTTGAAGTTCAG |
| pDPG004, 005, 006_seq-r | ACAATGTCATTATTCAGATCAAGCGG |
| pDPG005_seq-f | ACTTCAAATCCTGCGTCAGTTGG |
| pDPG005_seq-r | TGTGTCAAATGTATATTGTTTGGTACGC |
| US_1kb-us-*ccmG*_seq-f | ACGGGAGAACGTCAGGTGAG |
| DS_1kb-ds-*ccmG*_seq-r | TGCCGGTGAAACTTCCTGTATTTG |
| US_1kb-us-*ccdA*-seq-f | TCCATAGCAAGGGCTCTGTCAC |
| DS_1kb-ds-*ccdA*-seq-r | TCGTAGCAATCGCAAGGGAAAGTG |
| *ccmABC*-f for pDPG014 | **AATAAATTAAGGAGGAAATTCA**ATGGAAAACGCGGTCTCCATAAG |
| *ccmABC*-r for pDPG014 | **TTTTAAAACCCCGTATGGCTCG**TTATGTAACACAAATTGAAAAAACACAC |
| *ccmE*-f for pDPG014 | **TTTTTCAATTTGTGTTACATAA**CGAGCCATACGGGGTTTTAAAATG |
| *ccmE*-r for pDPG014 | **ATTAAATGGGGAATAATAGTTT**TTACTCCGTATACTTTGACGGGC |
| *ccmF_1_F_2_*-f for pDPG014 | **CCGTCAAAGTATACGGAGTAA**AAACTATTATTCCCCATTTAATTG |
| *ccmF_1_F_2_*-r for pDPG014 | **TACATTATACGAAGTTATCAAGA**TCATTCCTCGCTAAAATACTTC |
| P*serC*-f for pDPG015 | **AGCCTTTTTTTTTCGAAGTTT**GATCTCAACCACCTTTTTTC |
| P*serC*-r for pDPG015 | **TAACCTATTCATAATGACCAC**ATCTTTTCCTTTTTTGGTACTG |
| *mmcA*-TAP-f for pDPG015 | **AGTACCAAAAAAGGAAAAGAT**GTGGTCATTATGAATAGGTT |
| *mmcA*-TAP-r for pDPG015 | **TAAAGTGTTTGATTTATTGAGT**TTAACCTGATTTTTCAAACTG |
| T*mcr*(Ma)-f for pDPG015 | **CCTCAGTTTGAAAAATCAGGTTAA**ACTCAATAAATCAAACACTTTAAAAC |
| T*mcr*(Ma)-r for pDPG015 | **GCCGGCGCGCCTGCAGGTTT**GATTTCCGGAAAAACAGTAAAG |
| pDPG014_seq-r1 | TCCTTCGGCAAGATTAGAAG |
| pDPG014_seq-f1 | CTTCTAATCTTGCCGAAGG |
| pDPG014_seq-r2 | TGCTTACATAAGCCGAACC |
| pDPG014_seq-f2 | TCGGCTTATGTAAGCACCG |
| pDPG014_seq-r3 | TGAAAAGATCTCGGTCTCC |
| pDPG014_seq-f3 | TCAATTATGAGGTCGGTTTCG |
| pDPG014_seq-r4 | AGTCCTCAAAACATGAGAAATC |
| pDPG014_seq-f4 | TCATGTTTTGAGGACTTCAGG |
| pDPG014_seq-r5 | ATCTCAGGCAGCAAATTTCC |
| pDPG014_seq-f5 | TAGGAAATTTGCTGCCTGAG |
| pDPG014_seq-r6 | ATCCTTTGCCCAGATCGAAC |
| pDPG014_seq-f6 | TCGATCTGGGCAAAGGATG |
| *ccmE*-f for pKES30, 38, 40 | **TGATTTTAATAAATTAAGGAGGAAATTCAT**ATGAATAAAAAAAAGAAATCACTGC |
| *ccmE*-r1 for pKES30 | **CAAACTGAGGATGTGACCACCCGCCACCCT**CCGTATACTTTGACGGGCA |
| *ccmE*-r2 for pKES30 | **GTCGTCGTCCTTGTAGTCCCCGCCACCT**TTTTCAAACTGAGGATGTGACC |
| *ccmE(C120A)-*r1 for pKES40 | **TTACTCCGTATACTTTGACGGTGC**TCCGGTAATGATCTTATTTGCC |
| *ccmE(C120H)*-r1 for pKES38 | **TTACTCCGTATACTTTGACGGGTG**TCCGGTAATGATCTTATTTGCC |
| *ccmE(C120A)*-r2 for pKES40 | **CAAACTGAGGATGTGACCACCCGCCACCCTC**CGTATACTTTGACGG**TGC** |
| *ccmE(C120H)*-r2 for pKES38 | **CAAACTGAGGATGTGACCACCCGCCACCCTC**CGTATACTTTGACGG**GTG** |
| *ccmE-*r3 for for pKES30, 38, 40 | **GCATACATTATACGAAGTTATCAAGATTAGAT**GTCGTCGTCCTTGTAGTC |
| pDN402_us_f | **TTTGGAGCCTTTTTTTTTCGAAGTTTAAAC**CGG TTG ACT ATG CAG GTA CG |
| pDN402_us_r | CAG TAG AAG CAG CAC AGC GA |
| pDN402_ds_f | **GTGTCGGGAGTCGCTGTGCTGCTTCTACTG**GTA ACT TAC GAT AAG CCA AGA CC |
| pDN402_ds_r | **GTGTAATTCGGCGCGCCTGCAGGTTTAAAC**GAC GGT ATT CTT AAC CAC AGG |
| DDN009_seq_f | CCT GTT TGG TCC AAT ACA TC |
| DDN009-seq_r | ACT TCA GCT TCT GTC TGG AC |
| *mmcA*_f for pDN409 | **TGATTTTAATAAATTAAGGAGGAAATTCAT**GTG GTC ATT ATG AAT AGG TT |
| *mmcA*_r for pDN409 | GAG TGC AGG CTC CGT TTC A |
| C_TAPtag_f for pDN409 | **CCTGTAGAAGTTGAAACGGAGCCTGCACTC**GAC GAT GAC GAC AAG GGT GG |
| C_TAPtag_r for pDN409 | **AGCATACATTATACGAAGTTATCAAGATTA**ACCTGATTTTTCAAACTGAGG |
| pDN409_seq_f | TGA GAT TTC CCT ATC AGT GAT |
| pDN409_seq_r | CTC ACT AAA GGG ATA ACT TCG |
| pDN412_us_f | **TTTGGAGCCTTTTTTTTTCGAAGTTTAAAC**CAC CGG TAA GAA AAG AAT CTG G |
| pDN412_us_r | **GGTAATGATCTTATTTGCCTCAAACGTAGA**AGC AAC TGC AAG CAG TGA TT |
| pDN412_ds_f | TCT ACG TTT GAG GCA AAT AAG |
| pDN412_ds_r | **GTGTAATTCGGCGCGCCTGCAGGTTTAAAC**CTA AAT GGT ATC ATT TGT GGC |
| DDN010_seq_f | CAA CTG AAA ATG ACT TTT CG |
| DDN010_seq_r | ATA CAC CTG ATA AAG TTA GTG |
| pDN424_us_f | TTG TGA AAT TGC TCA CGA AC |
| pDN424_us_r | GAG AAC ATC GCT TAG CTT TTT C |
| pDN424_ds_f | **TTTTTTATGAAAAAGCTAAGCGATGTTCTC**GCT CAG GTA AAC GGA ATC AA |
| pDN424_ds_r | **GTGTAATTCGGCGCGCCTGCAGGTTTAAAC**AAA GGT CGA GAA GAT GAC C |
| pDN425_us_f | **TTTGGAGCCTTTTTTTTTCGAAGTTTAAAC**CAG CAC CAT ATT TGC TAC AG |
| pDN425_us_r | TAT CCA GAT GAG TCC CAT TC |
| pDN425_ds_f | **ATGAATACTGGAATGGGACTCATCTGGATA**GTT CTC CTT GTC AGG AAG TA |
| pDN425_ds_r | **GTGTAATTCGGCGCGCCTGCAGGTTTAAAC**GTA AAG CCA ACA ACA ACT TCC |
| pDN426_ds_f | **GTTTTTTATGAAAAAGCTAAGCGATGTTCTC**GTT CTC CTT GTC AGG AAG TA |
| DDN011_012_013_seq_f | TTT TCG TCC AGC ACT TTT AC |
| DDN011_012_013_seq_r | TAC CAG GGT TCT TTC TTG AC |
| pDN444_us_f | **TTTGGAGCCTTTTTTTTTCGAAGTTTAAAC**GAG CAG GAA GAA AAG AGC TTG |
| pDN444_us_r | **TCCGGCAGATCTGCGTGCGTCAAGTTCCTC**TGA TAG TTC CCT TAT GGA GAC C |
| pDN444_ds_f | GAG GAA CTT GAC GCA CGC AG |
| pDN444_ds_r | **GTGTAATTCGGCGCGCCTGCAGGTTTAAAC**CGG CAT AAT CCG CTC TGA CTG |
| DDN029_seq_f | GGA GTT CCA GGT TAC AGA CTG |
| DDN029_seq_r | GTC TGG TGC TGA TTA TGG AC |
