# Supplementary material for: An Archaea-specific c-type cytochrome maturation machinery is crucial for methanogenesis in Methanosarcina acetivorans
Source: eLife. 2022 Apr 5;11:e76970. doi: 10.7554/eLife.76970 (PMC9084895; doi:10.7554/eLife.76970)
Supplement: Supplementary file 6. [file elife-76970-supp6.docx]

**Supplementary Table 6:** List of *Methanosarcina* strains used in this study

| Strains | Genotype | Construction details | Source |
| --- | --- | --- | --- |
| *M. acetivorans* strains | | | |
| WWM60 | Δ*hpt*::P*mcrB*-*tetR* | - | (Guss et al., 2008) |
| DDN009 | Δ*hpt*::P*mcrB*-*tetR*, Δ*mmcA* | WWM60 was transformed to Pur^R^ with pDN404; plasmid-cured strain was isolated by plating on medium with 8ADP | This study |
| DDN010 | Δ*hpt*::P*mcrB*-*tetR*, Δ*ccmE* | WWM60 was transformed to Pur^R^ with pDN414; plasmid-cured strain was isolated by plating on medium with 8ADP | This study |
| DDN011 | Δ*hpt*::P*mcrB*-*tetR*, Δ*ccmF_1_* | WWM60 was transformed to Pur^R^ with pDN430; plasmid-cured strain was isolated by plating on medium with 8ADP | This study |
| DDN012 | Δ*hpt*::P*mcrB*-*tetR*, Δ*ccmF_2_* | WWM60 was transformed to Pur^R^ with pDN431; plasmid-cured strain was isolated by plating on medium with 8ADP | This study |
| DDN013 | Δ*hpt*::P*mcrB*-*tetR*, Δ*ccmF_1_*Δ*ccmF_2_* | WWM60 was transformed to Pur^R^ with pDN432; plasmid-cured strain was isolated by plating on medium with 8ADP | This study |
| DDN016 | DDN009/ pDN409  [*PmcrB(tetO4)-mmcA- C- TAP tag*] | DDN009 was transformed to Pur^R^ with pDN409; isolates were verified by Sanger sequencing and grown in HS medium supplemented with 2 µg/mL Puromycin | This study |
| DDN029 | Δ*hpt*::P*mcrB*-*tetR*, Δ*ccmABC* | WWM60 was transformed to Pur^R^ with pDN446; plasmid-cured strain was isolated by plating on medium with 8ADP | This study |
| DDN033 | Δ*hpt*::P*mcrB*-*tetR*, Δ*ccmG* | WWM60 was transformed to Pur^R^ with pDPG007; plasmid-cured strain was isolated by plating on medium with 8ADP | This study |
| DDN034 | Δ*hpt*::P*mcrB*-*tetR*, Δ*ccdA* | WWM60 was transformed to Pur^R^ with pDPG008; plasmid-cured strain was isolated by plating on medium with 8ADP | This study |
| DDN035 | Δ*hpt*::P*mcrB*-*tetR*, Δ*ccmG*Δ*ccdA* | WWM60 was transformed to Pur^R^ with pDPG009; plasmid-cured strain was isolated by plating on medium with 8ADP | This study |
| DDN037 | DDN009/pDPG010  [[*PmcrB(tetO4)-uidA*] | DDN009 was transformed to Pur^R^ with pDPG010; isolates were verified by Sanger sequencing and grown in HS medium supplemented with 2 µg/mL Puromycin | This study |
| DDN038 | WWM60/pDPG010  [*PmcrB(tetO4)-uidA*] | WWM60 was transformed to Pur^R^ with pDPG010; isolates were verified by Sanger sequencing and grown in HS medium supplemented with 2 µg/mL Puromycin | This study |
| DDN039 | WWM60/pDN409  [*PmcrB(tetO4)-mmcA-*C- TAP tag] | WWM60 was transformed to Pur^R^ with pDN409; isolates were verified by Sanger sequencing and grown in HS medium supplemented with 2 µg/mL Puromycin | This study |
| DDN040 | DDN010/pDN409  [*PmcrB(tetO4)-mmcA-* C- TAP tag] | DDN010 was transformed to Pur^R^ with pDN409; isolates were verified by Sanger sequencing and grown in HS medium supplemented with 2 µg/mL Puromycin | This study |
| DDN041 | DDN029/pDN409  [*PmcrB(tetO4)-mmcA-*C-TAP tag] | DDN029 was transformed to Pur^R^ with pDN409; isolates were verified by Sanger sequencing and grown in HS medium supplemented with 2 µg/mL Puromycin | This study |
| DDN047 | DDN013/pDN409  [*PmcrB(tetO4)-mmcA-*C-TAP tag] | DDN013 was transformed to Pur^R^ with pDN409; isolates were verified by Sanger sequencing and grown in HS medium supplemented with 2 µg/mL Puromycin | This study |
| DDN052 | DDN012/pDN409  [*PmcrB(tetO4)-mmcA-*C-TAP tag] | DDN012 was transformed to Pur^R^ with pDN409; isolates were verified by Sanger sequencing and grown in HS medium supplemented with 2 µg/mL Puromycin | This study |
| DDN054 | DDN011/pDN409  [*PmcrB(tetO4)-mmcA-*C-TAP tag] | DDN011 was transformed to Pur^R^ with pDN409; isolates were verified by Sanger sequencing and grown in HS medium supplemented with 2 µg/mL Puromycin | This study |
| DDN060 | DDN033/pDN409  [*PmcrB(tetO4)-mmcA-*C-TAP tag] | DDN033 was transformed to Pur^R^ with pDN409; isolates were verified by Sanger sequencing and grown in HS medium supplemented with 2 µg/mL Puromycin | This study |
| DDN062 | DDN034/pDN409  [*PmcrB(tetO4)-mmcA-*C-TAP tag] | DDN034 was transformed to Pur^R^ with pDN409; isolates were verified by Sanger sequencing and grown in HS medium supplemented with 2 µg/mL Puromycin | This study |
| DDN064 | DDN035/pDN409  [*PmcrB(tetO4)-mmcA-*C-TAP tag] | DDN035 was transformed to Pur^R^ with pDN409; isolates were verified by Sanger sequencing and grown in HS medium supplemented with 2 µg/mL Puromycin | This study |
| DDN078 | DDN010/pKES31  [*PmcrB(tetO4)-ccmE-*C-TAP^m^ tag] | DDN010 was transformed to Pur^R^ with pKES031; isolates were verified by Sanger sequencing and grown in HS medium supplemented with 2 µg/mL Puromycin | This study |
| DDN091 | DDN029/pKES31  [*PmcrB(tetO4)-ccmE-*C-TAP^m^ tag] | DDN029 was transformed to Pur^R^ with pKES031; isolates were verified by Sanger sequencing and grown in HS medium supplemented with 2 µg/mL Puromycin | This study |
| DDN092 | DDN010/pKES39  [*PmcrB(tetO4)-ccmE*(C120H*)-*C*-*TAP^m^ tag] | DDN010 was transformed to Pur^R^ with pKES039; isolates were verified by Sanger sequencing and grown in HS medium supplemented with 2 µg/mL Puromycin | This study |
| DDN093 | DDN010/pKES41  [*PmcrB(tetO4)-ccmE*(C120A*)-*C-TAP^m^ tag] | DDN010 was transformed to Pur^R^ with pKES041; isolates were verified by Sanger sequencing and grown in HS medium supplemented with 2 µg/mL Puromycin | This study |
| DDN099 | WWM60/ pKES31  [*PmcrB(tetO4)-ccmE-*C-TAP^m^ tag] | WWM60 was transformed to Pur^R^ with pKES031; isolates were verified by Sanger sequencing and grown in HS medium supplemented with 2 µg/mL Puromycin | This study |
|  |  |  |  |
| *M. barkeri* strains | | | |
| WWM155 | Δ*hpt*:: *PmcrB-tetR-phiC31int-attP* | - | (Guss et al., 2008) |
| DDN085 | WWM155/pJK029A  [*PmcrB(tetO4)-uidA*] | WWM155 was transformed to Pur^R^ with pJK029; isolates were verified by Sanger sequencing and grown in HS medium supplemented with 2 µg/mL Puromycin | This study |
| DDN086 | WWM155/pDPG014  [*PmcrB(tetO4) -(ccmABCEF_1_F_2_)_Ma_*] | WWM155 was transformed to Pur^R^ with pDPG014; isolates were verified by Sanger sequencing and grown in HS medium supplemented with 2 µg/mL Puromycin | This study |
| DDN087 | WWM155/pDPG015  [*PserC-(mmcA)_Ma_-*C-TAP tag] | WWM155 was transformed to Pur^R^ with pDPG015; isolates were verified by Sanger sequencing and grown in HS medium supplemented with 2 µg/mL Puromycin | This study |
| DDN088 | WWM155/pDPG017  [*PserC-(mmcA)_Ma_-*C-TAP tag*, PmcrB(tetO4)-(ccmABCEF_1_F_2_)_Ma_*] | WWM155 was transformed to Pur^R^ with pDPG017; isolates were verified by Sanger sequencing and grown in HS medium supplemented with 2 µg/mL Puromycin | This study |
